# Centrosome aberrations in human mammary epithelial cells driven by cooperative interactions between p16^INK4a^ deficiency and telomere-dependent genotoxic stress

**DOI:** 10.18632/oncotarget.4958

**Published:** 2015-07-22

**Authors:** Daniel Domínguez, Purificación Feijoo, Aina Bernal, Amaia Ercilla, Neus Agell, Anna Genescà, Laura Tusell

**Affiliations:** ^1^ Cell Biology Unit, Department of Cell Biology, Physiology and Immunology, Bioscience School, Universitat Autònoma de Barcelona, Bellaterra, Spain; ^2^ Departament de Biologia Cellular, Immunologia i Neurociències, Facultat de Medicina, Universitat de Barcelona, IDIBAPS, Barcelona, Spain

**Keywords:** centrosome aberrations, CDKN2A, human mammary epithelial cells, telomere dysfunction

## Abstract

Virtually all human cancers display chromosome instability (CIN), a condition in which chromosomes are gained or lost at a high rate. CIN occurs early in cancer development where it may undermine the advance of the neoplastic disease. With the aim of establishing the mechanisms underlying CIN in cancer, we investigated possible links between telomere-dysfunction and centrosome defects, which were seen to coincide in early in breast carcinogenesis using human mammary epithelial cells (HMECs). In this study, we show that *TP53* proficient vHMECs cells develop centrosome aberrations when telomere-dysfunction genotoxic stress is produced in the presence of a defective p16^INK4a^ setting and in parallel with an activation of the DNA damage checkpoint response. These aberrations consist of the accumulation of centrosomes in polyploid vHMECs, plus centriole overduplication in both diploid and polyploid cells, thus reflecting that distinct mechanisms underlie the generation of centrosome aberrations in vHMECs. Transduction of vHMEC with hTERT, which rescued the telomere dysfunction phenotype and consequently reduced DNA damage checkpoint activation, led to a progressive reduction of centrosome aberrations with cell culture, both in diploid and in polyploid vHMECs. Radiation-induced DNA damage also raised centrosome aberrations in vHMEC-hTERT. Collectively, our results, using vHMECs define a model where p16^INK4a^ deficiency along with short dysfunctional telomeres cooperatively engenders centrosome abnormalities before p53 function is compromised.

## INTRODUCTION

Chromosomal instability (CIN) is a hallmark of almost all human cancer cells, and reflects ongoing changes of chromosome structure and number over time. CIN most likely occurs early in the development of cancer, and may represent an important step in promoting the multiple genetic changes required for the initiation and/or progression of the disease. By constantly reshuffling the cell karyotype, CIN is thought to confer a selective advantage on subclones of cells, enabling their outgrowth and eventual dominance in a local tissue environment. To date, however, the specific molecular mechanisms that underlie CIN in cancer cells still remain obscure.

Centrosomes function as microtubule-organizing centers of animal cells that build up the mitotic spindle during cell division. Therefore, the acquisition of extra centrosomes in a cell can arrange aberrant spindles that, through the formation of merotelic attachments, may lead to the onset of CIN [1-3]. Emerging data are demonstrating the detection of centrosome defects in several pre-neoplasia lesions, but not in normal tissue (reviewed by [4]), hence, it has been hypothesized that centrosome amplification might lead to the malignant transformation of cells. Centrosome defects in human cancer cells consist of structural changes in shape, size, number, and position, as well as functional defects. Potential mechanisms for the generation of centrosome aberrations in cancer cells include alterations in proteins controlling the centrosome duplication cycle, which could initiate multiple cycles of centriole replication -overduplication -within a single cell cycle [5]; or accumulation of extra centrosomes when cell division does not occur because of mitotic slippage, endoreduplication, aborted cytokinesis or cell fusion [6, 7]. Another possible scenario for increasing numbers of centrosomes involves *de novo* formation of centrioles during interphase [8]. Although these fundamental processes are not mutually exclusive and could be acting at the same time or in a sequential fashion, the precise mechanisms driving centrosome aberrations early in cancer development are still undefined. Another possible cause for the onset of CIN in sporadic cancers is telomere dysfunction. When telomeres become dysfunctional, they set breakage-fusion-bridge (BFB) cycles in motion that are capable of producing high levels of CIN, generating both structural and numerical chromosome aberrations, as well as changes in cell ploidy [9, 10]. Very short telomeres have also been reported to be an early alteration in many human cancers [11, 12]. And compelling evidence, in mouse models, supports the notion that loss of telomere repeats contributes to carcinogenesis [13].

In breast cancer, there is evidence for the presence of centrosome aberrations -before *TP53* mutations are attained [14-16] -and high levels of end-to-end fusions [17] as early events in carcinogenesis. The aim of this study was to investigate whether there is a functional explanation for the coincident detection of telomere dysfunction and centrosome defects early in breast cancer development. For this reason, we used the human mammary epithelial cell model (HMEC), which mimics *in vitro* the genomic events driving malignant progression in the breast [18, 19]. When HMECs are grown in culture under standard conditions, they experience a growth plateau from which some cells can escape, proliferate, expand and display progressive telomere dysfunction due to *CDKN2A* promoter hypermethylation [20]. Considering that cells with p16^INK4a^ deficiencies develop centrosome aberrations when a transient inhibition of DNA synthesis occurs [21], we hypothesized that a similar phenotype could arise due to the genotoxic damage driven by telomere dysfunction. Accordingly, our study demonstrates the accumulation of centrosome aberrations, concomitant to the intensification of the telomere-dysfunction phenotype, and in parallel with an activation of the DNA damage checkpoint response in vHMECs. Moreover, transduction of vHMEC with hTERT, which rescues the telomere dysfunction phenotype and consequently reduced DNA damage checkpoint activation, rendered a progressive reduction of centrosome aberrations with cell culture. Noteworthy, in contrast to the centriole pair splitting events reported [21] the main centrosomal aberration in telomere compromised p16^INK4a^ -deficient vHMECs was the presence of centriole overduplication. We show that the loss of p16^INK4a^ function in vHMEC alone is not sufficient to cause centrosome amplification, but rather creates the permissive conditions for their development in response to the genotoxic stress of telomere dysfunction.

## RESULTS

### Tetraploid populations increase in telomere-deficient vHMECs

For the evaluation of ploidy levels in post-stasis vHMEC lines (830 and 440212) throughout the cell culture, a combination of β-tubulin immunofluorescence with fluorescent *in situ* hybridization (FISH) was performed. This immunoFISH protocol enabled the different nucleus inside the same cytoplasm to be visualized, allowing the ploidy of mononucleated (MN) and binucleated (BN) cells to be easily recorded. vHMEC were analyzed at an early culture stage (PD19 and PD21, for 830 and 440212, respectively) just after a period of selection when clones with p16^INK4a^ inactivation (Figure S1A) acquire proliferation capacity due to promoter hypermethylation [20, 22]. In addition, late culture stages (PD34, for both cell donors) were analyzed to detect any abnormalities gained over time. These specific vHMEC lines have a limited potential and cease proliferation -agonescence -at around PD35.

**Figure 1:**
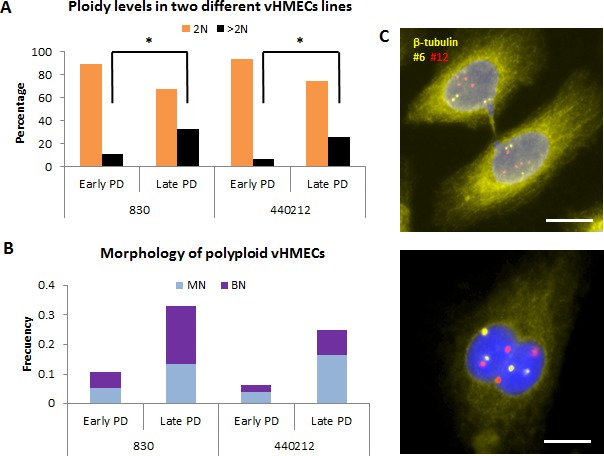
Tetraploidization events are promoted by telomere-dysfunction Variant human mammary epithelial cells (vHMECs) derived from distinct donors were analyzed for ploidy levels using β-tubulin antibodies in combination with oligonucleotide specific probes for the centromeric region of chromosome 6 and 12. **A.** vHMECs from donor 830 and 440212 were grown on chamber-slides at early and late PDs and processed with ImmunoFISH protocols. Tetraploidization events increase significantly with PDs in both, donor 830 and 440212. *P < 0.05 X^2^-test. **B.** The combined detection of β-tubulin and centromeric specific DNA probes allowed the morphology of polyploid cells to be distinguished. Polyploid cells were either mononucleated (MN) or binucleated (BN) and both types of cells increased with PDs with no prominent differences between the two types. **C.** Representative image of vHMECs after ImmunoFISH protocols. The top image shows two mononucleated polyploid cells after mitosis still connected by a cytoplasmic bridge. Each nucleus displays four copies of each centromeric-specific probe. The bottom image shows a binucleated polyploid cell. White bar represents 10 μm.

A total of 1566 cells (554 for 830, and 1012 for 440212), grown in chamberslides, were evaluated for ploidy levels using two different centromere-specific probes. At early PD, a small fraction of cells was confirmed to be polyploid in both donors. Importantly, however, there was a significant increase in polyploidy with PDs for both cell lines (10.66% *vs* 32.27% in 830, X^2^, *p* < 0.05; and 6.44% *vs* 25.26% in 440212, X^2^, *p* < 0.05) (Figure 1A). These results are in accordance with the already defined tetraploidization effect of telomere dysfunction [9, 23]. Indeed, signatures of telomere dysfunction such as telomere-signal free ends, chromosome end-to-end fusions without detectable telomeric DNA and/or anaphase bridges were observed to increase with PDs in both cell lines (Figure S1B, S1C). In addition, the morphological characterization of the vHMEC populations made it possible to determine whether the polyploid fraction of cells was made up of MN and/or BN cells (Figure 1B, 1C). Both types of polyploid populations increased with PDs in each vHMEC cell line without either being predominant (X^2^, *p* = 0.638 in 830; X^2^, *p* = 0.167 in 440212). Therefore, these results indicate that the intensification of the telomere-dysfunction phenotype in vHMECs engenders increasing polyploid populations that most likely arise through both cytokinesis failure and bypass of mitosis and endoreduplication.

### Centrosome aberrations increase -both in number and in size -in diploid and polyploid vHMECs

**Figure 2:**
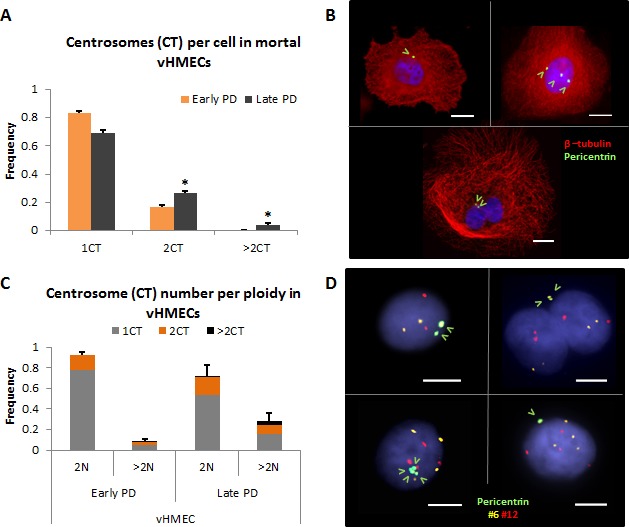
Centrosome aberrations in vHMECs increase with PDs **A.** The number of centrosomes per cell and throughout the PDs was analyzed using β-tubulin and the centrosome marker pericentrin. At early PD, vHMECs contained mostly one centrosome (1CT) or two centrosomes (2CT) per cell. vHMECs with more than 2 centrosomes (> 2CT) are virtually recorded at late PD. Error bars, standard error of the mean; *n* = 2, *P < 0.05 X^2^-test (early PD *vs.* late PD). **B.** Representative micrographs of pericentrin and β-tubulin by immunofluorescence. Mononucleated cell with one pericentrin signal (upper left) and with three pericentrin signals (upper right) and a binucleated cell showing two pericentrin signals (bottom). Pericentrin signals are pointed with open arrows. DNA is counterstained with DAPI. Bar represents 10 μm. **C.** The use of immunoFISH protocols allowed for the accurate analysis of centrosome numbers in vHMECs according to their ploidy status. Abnormal centrosome numbers, more than 2CT, prevailed in polyploid vHMECs rather than in diploid. But strikingly, more than half of polyploid vHMECs at late PD contained only one pericentrin spot. Error bars, standard error of the mean; *n* = 2. **D.** Representative images of vHMECs after immunoFISH of pericentrin antibodies and centromeric probes for chromosome 6 and 12. Mononucleated diploid cell with 2 CT (upper left), binucleated tetraploid cell with 2 CT (upper right), mononucleated tetraploid cell with 4CT (bottom left), and mononucleated tetraploid cell with only 1 CT (bottom right). Pericentrin signals are pointed with open arrows and DNA is counterstained with DAPI. White bar on micrographs represents 5 μm. In this analysis, results of both cell lines were plotted as no statistical differences between them were observed.

The emergence of polyploid populations throughout the cell culture would lead to the presence of cells with supernumerary centrosomes. Therefore, we assessed and quantified for the presence of centrosome aberrations in vHMEC. Immunofluorescence using antibodies specific for β-tubulin and pericentrin, a core component of the pericentriolar protein rich matrix (PCM), was performed throughout the cell culture. Centrosome numbers vary during the cell cycle: at G1, cells contain a single centrosome that duplicates once the cell enters S-phase; the duplicated centrosomes mature during G2; and at the onset of mitosis separate to form the bipolar spindle. Therefore, a cell containing more than two centrosomes, i.e. more than two pericentrin dots per cell, should be considered abnormal. The mean number of pericentrin signals per cell at early PD stages was 1.17 and this rate significantly increased to 1.45 in late PDs (U-Mann Whitney, *p* < 0.05). Even though cells carrying > 2CT significantly increased with PDs (0.4% on PD21 up to 4.4% on PD34, X^2^, *P* < 0.05), they were few in number and seen mainly at late PD (Figure 2A, 2B).

Given that telomere-dysfunction engenders polyploid cells, it was of relevance to ascertain whether mononucleated cells were diploid and contained a correct number of centrosomes, i.e. 1 or 2, or instead were polyploid and thus showed an aberrant number of centrosomes per cell. Therefore, we performed an immunoFISH protocol combining pericentrin antibodies with centromere-specific oligoFISH probes to evaluate centrosome aberrations in the diploid and polyploid fraction of cells (Figure S2). Throughout the cell culture most diploid cells displayed either 1 or 2 centrosomes; only 3 out of 472 diploid cells presented more than two centrosomes at late PD (Figure 2C, 2D). Notwithstanding, the most surprising observation was that more than half of the polyploid population of cells (69% at early PD and 56% at late PD) presented only one centrosome (Figure 2C, 2D). As polyploid cells should present at least 2 CT, except in the case of clustering of centrosomes or extrusion of one centrosome outside the cell, centrosome size aberrations were also quantified in vHMECs.

**Figure 3:**
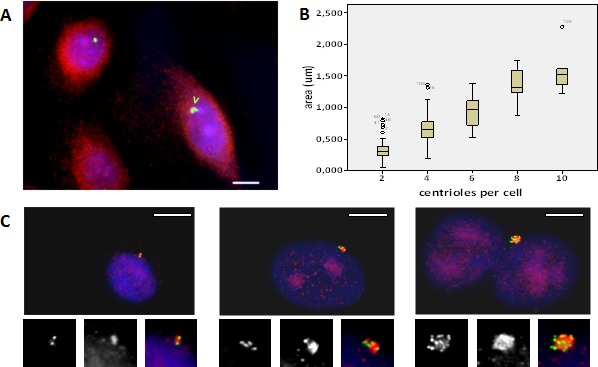
Large centrosomes, indicative of centriole amplification events, are observed in vHMECs Immunofluorescence protocols demonstrated the presence of large centrosomes. **A.** The micrograph shows two mononucleated cells after pericentrin and β-tubulin staining. The cell on the right side displays—pointed arrow—a large pericentrin signal, bigger than twice the pericentrin signal of the upper left side cell. The bar represents 5 μm. **B.** The use of antibodies against pericentrin and centrin in vHMEC-hTERT determined that pericentrin area size became greater with an increasing number of centrioles. **C.** Pericentrin and centrin immunofluorescence, of three cells showing increasing numbers of centrin dots in green: 2 centrin signals (left side), 4 centrin signals (middle), and > 10 centrin signals (right side), and pericentrin area size in red. At the bottom of each image, insets showing individual centrin, pericentrin and merged respective images. Bar represents 5 μm.

Abnormally large centrosomes, i.e. showing a pericentrin signal that was more than twice their usual size, were observed in vHMECs (Figure 3A). Significantly, these aberrantly sized centrosomes were observed both in diploid and polyploid vHMEC populations at a higher incidence than numerical centrosome aberrations (Table 1). And their frequency significantly increased with PDs, both in diploid (6.59% up to 11.35% on 830, X^2^, *p* < 0.05; 4.87% up to 9.04% on 440212, X^2^, *p* < 0.05) and in polyploid vHMECs (2.93% up to 13.83% on 830, X^2^, *p* < 0.05; 2.67% up to 16.22% on 440212, X^2^, *p* < 0.05) (Table 1). In order to determine if these abnormally large pericentrin signals corresponded to centriole amplification events rather than a normal centrosome matrix enlargement [24], immunofluorescence using antibodies against pericentrin and centrin, which label single centrioles, was performed in vHMEC-hTERT. After analyzing 114 centrosomes, it was found that a size increase in the pericentrin labeling area was directly related to an increase of centrioles per centrosome. The area of a pericentrin signal containing 2 or 4 centrioles had a mean size of 0.468±0.28 μm^2^, whereas centrosomes with more than 4 centrioles had a mean pericentrin area of 1.272±0.41 μm^2^ (ANOVA, *P* < 0.05) (Figure 3B, 3C). Therefore, these results support that an enlargement in the size of the pericentrin labeling area is directly related to a higher number of centrioles per centrosome.

**Table 1:** Size centrosome aberrations in diploid and polyploid vHMECs

		Large CT		
		2N	>2N	***TOTAL***
830vHMEC	**Early**	6.59%	2.93%	*9.52%*
	**Late PD**	11.35%	13.83%	*25.17%*
440212vHMEC	**Early**	4.87%	2.67%	*7.47%*
	**Late PD**	9.04%	16.22%	*25.26%*

Overall, centrosome abnormalities significantly increased in vHMECs with PDs in parallel with the intensification of the telomere-dysfunction phenotype. These aberrations consisted of supernumerary, as well as oversized centrosomes. Importantly, large centrosomes, which were the predominant type of centrosome aberrations in vHMECs, could account for an excess number of centrioles per centrosome.

### vHMECs acquire supernumerary centrioles throughout the cell culture

**Figure 4:**
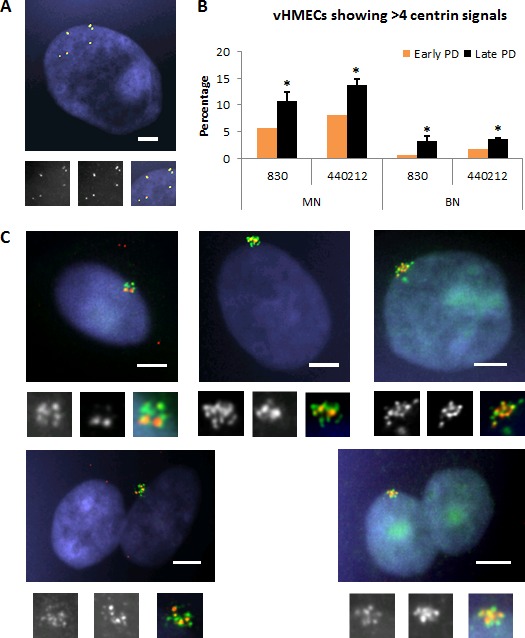
Amplification of centrioles occurs in mononucleated and binucleated vHMECs, and increases with PDs **A.** Centriole splitting, where centrin dots co-stained with NEDD1 are dispersed throughout the cytoplasm, occurs in vHMECs. At least 8 centrin/NEDD1 co-stained marks are observed in this cell, which consist of four centrin/NEDD1 singlets and two centrin/NEDD1 doublets. At the bottom of the image, insets show individual centrin, NEDD1 and merged image. Bar, 5 μm. **B.** Amplification of centrin—more than four spots—increases with PDs in both mononucleated and binucleated vHMECs. Error bars, standard error of the mean; *n* = 2, *P < 0.05 X^2^-test (early PD *vs.* late PD). **C.** Representative images of amplification of centrioles. On the first row, mononucleated vHMECs with > 4 centrin signals in combination with: 2 NEDD1 signals (left side), 4 NEDD1 signals (center) and > 4 NEDD1 signals (right side). On the second row, binucleated vHMECs with > 4 centrin signals in combination with: 4 NEDD1 (left side) or > 4 NEDD1 signals (right side). At the bottom of each image, insets show individual centrin and NEDD1 signals and the merged image. Bar, 5 μm.

Considering that increased centrosome volume and supernumerary centrioles are observed in breast carcinogenesis [3, 25], we sought to confirm whether centriole amplification events occurred in p16^INK4a^-deficient vHMECs. To do this, immunofluorescence using antibodies against centrin were combined with NEDD1 (neural precursor cell expressed developmentally downregulated gene 1), a protein component of the PCM that forms ring-shaped patterns around the mother and, to a lesser extent, the daughter centriole [26]. The analysis of 3147 cells, allowed identifying the most frequent NEDD1/centrin staining in MN and BN vHMECs (Table 2 and Figure S3). Usually all NEDD1/centrin signals were morphologically in close association, but some fragmentation of the centrosome, where single co-stained dots of NEDD1/centrin were spread throughout the cytoplasm, was also observed (Figure 4A). Moreover, and in agreement with [21] centrosome splitting significantly increased with PDs (0.09% *vs* 3.75% on 830, X^2^, *p* < 0.05; 0.20% *vs* 1.83% on 440212, X^2^, *p* < 0.05), albeit it was not the most frequent centrosome abnormality in vHMECs throughout the PDs. Centriole splitting in telomere-compromised vHMEC usually affected all centriole pairs and was demonstrated by huge flat cells that were probably senescent. This fits in with other studies that have reported an increase in supernumerary centrosomes, most likely due to centrosome fragmentation, coincident with the entry of late passage MEFs into senescence [27].

**Table 2:** NEDD1/centrin staining in vHMECs

		Early PD				Late PD			
		830		440212		830		440212	
		*n*	%	*n*	%	*n*	%	*n*	%
MONONUCLEATED	2 NEDD1/2 Centrin	767	67.52%	636	62.85%	111	36.04%	361	52.24%
	2 NEDD1/4 Centrin	39	3.43%	240	23.72%	46	14.94%	96	13.89%
	4 NEDD1/4 Centrin	230	20.25%	17	1.68%	71	23.05%	91	13.17%
	SUBTOTAL	1036	91.20%	893	88.24%	228	74.03%	548	79.31%
	2 NEDD1/> 4 Centrin	10	0.88%	61	6.03%	2	0.65%	47	6.80%
	4 NEDD1/>4 Centrin	19	1.67%	12	1.19%	14	4.55%	25	3.62%
	>4 NEDD1/>4 Centrin	36	3.17%	10	0.99%	17	5.52%	24	3.47%
	SUBTOTAL	65	5.72%	83	8.20%	33	10.71%	96	13.89%
	**MN TOTAL**	**1101**	**96.92%**	**976**	**96.44%**	**261**	**84.74%**	**644**	**93.20%**
BINUCLEATED	4 NEDD1/4 Centrin	28	2.46%	19	1.88%	37	12.01%	22	3.18%
	SUBTOTAL	28	2.46%	19	1.88%	37	12.01%	22	3.18%
	4 NEDD1/>4 Centrin	1	0.09%	14	1.38%	1	0.32%	11	1.59%
	>4 NEDD1/>4 Centrin	6	0.53%	3	0.30%	9	2.92%	14	2.03%
	SUBTOTAL	7	0.62%	17	1.68%	10	3.25%	25	3.62%
	**BN TOTAL**	**35**	**3.08%**	**36**	**3.56%**	**47**	**15.26%**	**47**	**6.80%**
		1136		1012		308		691	

Noteworthy, the main centrosomal aberration observed was the presence of centrin amplification events, i.e. more than 4 centrin signals per cell, which increased significantly with PDs (0.063 of cells at early PD to 0.140 at late PD on 830, X^2^, *p* < 0.05; and 0.099 of cells at early PD to 0.175 at late PD on 440212, X^2^, *p* < 0.05) (Table 2 and Figures 4B, 4C). Intensification of centrin amplifications events also increased throughout the cell culture for both MN and BN vHMECs (X^2^, *p* < 0.05, for both cell lines; Table 2). Collectively, whereas the observation of amplification events in BN cells established that this phenomenon occurred in the tetraploid fraction of vHMECs, it was not clear whether centrin amplification events also occurred in the diploid fraction of cells, as MN cells with different ploidy content coexist throughout the vHMEC culture.

**Figure 5:**
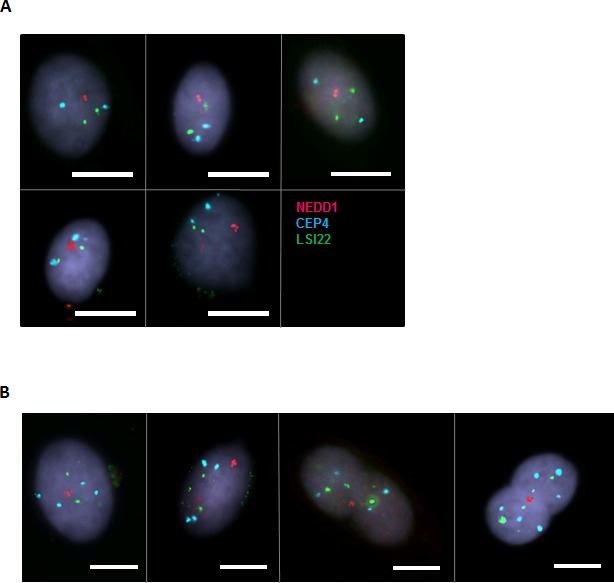
Cells showing 4 NEDD1 signals are either diploid or polyploid ImmunoFISH protocol of NEDD1 (red) antibody and DNA specific probes CEP4 -aqua- and LSI22 -green-, were combined to determine the DNA content of mononucleated cells with 2 and 4 NEDD1 signals. **A.** Representative images of diploid vHMECs with two (top side) and four (bottom side) NEDD1 signals. Scale bar represents 10 μm. **B.** Images show binucleated and mononucleated tetraploid vHMEC cells with 4 NEDD1 signals. Scale bar represents 10 μm.

At that point, a direct assessment to establish whether MN vHMECs showing centrin amplification events were either diploid or tetraploid in nature was difficult to obtain as it was not feasible to combine centrin staining with *in situ* hybridization. However, immunoFISH analysis of NEDD1 combined with two DNA probes demonstrated that MN cells showing 2 NEDD1 signals were diploid (47/47) whereas MN cells showing 4 NEDD1 signals were either diploid (10/25) or polyploid (15/25) (Figure 5A, 5B). Of relevance, these observations, together with the NEDD1/centrin staining patterns (Figure S3), revealed that similarly to centrin, NEDD1 protein also duplicates during the cell cycle and does it slightly later. Moreover, it indicated that cells containing 2 NEDD1 signals are truly diploid; cells with 4 NEDD1 signals are either diploid cells at late S/G2 cell cycle phase −2n^2c^ DNA content- or tetraploid cells in G1/early S-phase −4n^c^ DNA content-; and that cells containing > 4NEDD1 signals should be tetraploid cells at late S/G2 −4n^2c^ DNA content- or octoploid cells in G1/early S-phase −8n^c^ DNA content. As a whole, considering that amplification of centrioles is observed in cells containing 2 NEDD1, 4 NEDD1 and > 4 NEDD1 signals (Table 2), these results support the view that amplification of centrioles occurs in both diploid and polyploid vHMECs.

Another relevant question was to understand why, among the fraction of cells with amplified centrioles, only MN cells showing 4 NEDD1 signals significantly increased with PDs despite that the intensification of centrin amplification events occurred throughout the cell culture for both MN and BN vHMECs (Table 2). It must be considered that cells with 4 NEDD1 signals are a mixture of diploid and tetraploid cells. So that when a given diploid cell with amplified centrosomes duplicates NEDD1 protein, it raises the frequency of MN cells harboring 4 NEDD1 signals. Then, if this cell divides properly, the increased number of centrioles will be distributed into the two resulting daughter cells, thus diluting the amplified phenotype in the diploid with 2 NEDD1 signals fraction of cells. In contrast, if division does not occur, amplification events will accumulate with PDs in the tetraploid with 4 NEDD1 signals fraction of cells, giving their low mitotic index.

### Both centriole accumulation and overduplication account for extremely large centrosomes

To get further insight into the process responsible for centriole aberrations in vHMECs, it was important to establish whether centriole amplification emerged from a deregulation of the centriole replication cycle, i.e. centriole overduplication, or through accumulation of centrioles due to tetraploidization events. For that reason, we took advantage of the CEP170 antibody, a protein that localizes to the mother centriole sub-distal appendages [28] in combination with centrin. Using these antibodies, genuine centriole overduplication can be distinguished from centriole accumulation using the ratio between immature and mature centrioles (Figure S4).

**Figure 6:**
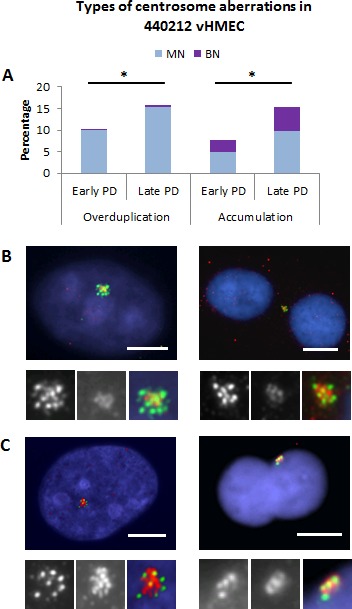
Centriole overduplication and accumulation increase in vHMEC with PDs By double staining of centrin antibodies—green—which labels all centrioles, and CEP170 antibodies—red—which labels mother centrioles, it was possible to evaluate the possible mechanisms accounting for centriole amplification events. **A.** Overduplication of centrioles was mainly seen in mononucleated -MN- vHMECs. In contrast, accumulation of centrioles was observed in both MN and binucleated -BN- vHMECs. * *P* < 0.05 X^2^-test, (early PD *vs.* late PD). **B.** Representative images of centriole overduplication: > 4 centrin signals together with 1 or 2 CEP170 spots. The MN cell—on the left side—displays 8 centrin signals and two CEP170. The BN vHMEC—on the right side—displays at least 8 centrin signals and 2 CEP170 spots. At the bottom of each image, insets show individual centrin and CEP170 signals and the merged image. **C.** Representative images of centrosome accumulation events. In MN cells, accumulation of centrosomes was considered when the cells displayed > 4 centrin signals and at least 50% of them were CEP170 labeled. This is the case for the vHMEC on the left side, showing 7 centrin signals. In BN cells, accumulation of centrosomes was considered when 4 centrin signals together with 2 CEP170 were recorded. An example of a BN vHMEC with accumulation of centrosomes is observed on the right side. At the bottom of each image, insets show individual centrin and CEP170 signals and the merged image. Bar represents 5 μm.

Quantification of CEP170/centrin staining in 440212 vHMEC cell line demonstrated that both accumulation and overduplication of centrioles were taking place throughout the cell culture (Figure 6A). Centriole overduplication affected mainly MN vHMECs and their incidence increased significantly with PDs (9.99% at early PD vs 17.86% at late PD, X^2^, *p* < 0.05). BN cells with centriole overduplication were few and no statistical increase was observed throughout the cell culture (X^2^, *p* = 0.702) (Figure 6A, 6B). Accumulation of centrioles affected both MN and BN cells, and intensification of this event was observed throughout the PDs (4.85% vs 10.88% for MN and 2.95% vs 6.66% for BN, X^2^, *p* < 0.05 for both types of cells). These results support the view that, in vHMECs, the emergence of polyploid cells throughout the cell culture leads to the accumulation of centrosomes with PDs. However, the presence of centriole overduplication events in MN cells suggests that other processes apart from tetraploidization might instigate centrosome abnormalities in vHMECs. In this regard, it has been determined that the suppressed expression of p16^INK4a^ in different cell types is coupled to centrosome dysfunction [21]. To corroborate this, we tested whether p16^INK4a^-proficient pre-stasis HMECs isolated from mammary tissue did present a centrosome-deficient phenotype. Centriole aberrations were evaluated after CEP170/centrin staining in pre-stasis HMECs at PD3 and compared to the incidence observed in p16^INK4a^-deficient 440212 vHMECs at early PD (PD21). Notably, a significant increase in centriole overduplication was observed in those cells where p16^INK4a^ was compromised (10.1% vs 2.4%, in post-stasis 440212 vHMEC and pre-stasis HMEC, respectively; X^2^, *p* < 0.05). Therefore, these data further confirm that loss of p16^INK4a^ in HMECs plays a causal role in centrosome dysfunction.

### Centrosome abnormalities decrease over time when telomere damage is abolished

**Figure 7:**
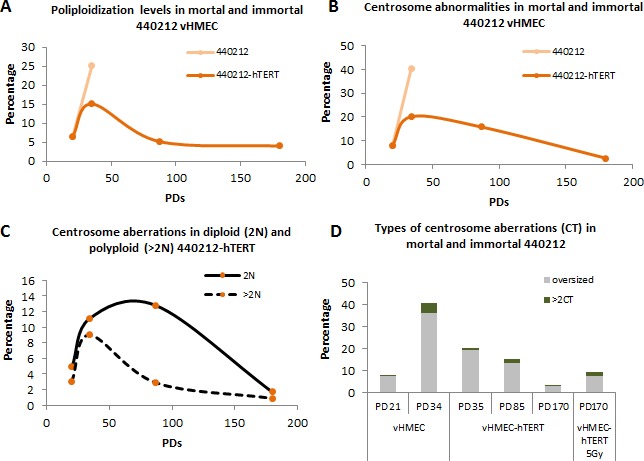
Restoration of telomere length reduces centrosome aberrations both in diploid and polyploid vHMECs-hTERT Immortalization of vHMECs was accomplished at early PD by transducing the cells with hTERT **A.** Evolution of polyploidization events in mortal cells - vHMECs - and immortal cells - vHMEC-hTERT - throughout the PDs. Transduction of vHMECs with hTERT concluded with the reduction of the polyploid fraction of cells because of suppression of the telomere-dysfunction phenotype and their compromised viability. **B.** Centrosome aberrations were followed during culture expansion of vHMEC and vHMEC-hTERT by means of β-tubulin and pericentrin immunofluorescence. Note that centrosome aberrations decline over PDs when chromosome ends are capped. **C.** ImmunoFISH protocols allowed a decline of centrosome aberrations to be determined in diploid and polyploid vHMEC-hTERT. **D.** DNA damage in the form of telomere-dysfunction increased centrosome aberrations in p16^INK4a^-deficient vHMECs. Telomerase reactivation and capping of telomere DNA free ends resulted in a progressive reduction of centrosome aberrations. The induction of DSBs in vHMEC-hTERT, through exposure to ionizing radiation, increased the centrosome aberrations again in vHMECs.

To delineate p16^INK4a^ deficiency as the only factor responsible for the generation of centrosome aberrations, vHMECs were immortalized with hTERT to minimize the telomere-dysfunction phenotype. vHMECs were transduced at early PD and their immediate and long effects on ploidy status and centrosome numbers and size were assessed. The restoration of telomerase function stabilizes the chromosome ends and thus might attenuate the generation of tetraploid cells. Accordingly, both vHMEC-hTERT cell lines showed telomeric FISH signals at the tips of all chromosomes and there was no evidence of telomere-dependent instability in the form of dicentric chromosomes at long times post-transduction (Figure S1B). Moreover, the analysis of cell ploidy with oligoFISH probes in vHMEC-hTERT grown in chamberslides showed a significant reduction of polyploid cells with increasing PDs, from 15.08% at PD35 to 4.04% at PD180 in 440212-hTERT, X^2^, *p* < 0.05, (Figure 7A) and from 10.83% at PD35 to 0.58% at PD132 in 830-hTERT, X^2^, *p* < 0.05 (Figure not shown). Particularly, in 440212 transduced cells, a slight increase was observed in the fraction of polyploidy cells 15 PDs after infection, thus indicating that once telomerase function is restored, there is a lag time before chromosome ends become capped (Figure 7A).

It was then assessed whether reactivation of telomerase and reduction of telomere-dependent damage lead in turn to a significant reduction of centrosome abnormalities in the transduced vHMEC-hTERT throughout the culture. Pericentrin staining in 440212 immortalized vHMEC depicted a reduction of centrosome aberrations, in number and in size, with PDs following telomerase reactivation and after an initial lag phase (Figure 7B). Importantly, when centrosome aberrations were distributed according to cell ploidy status, it was determined that the polyploid fraction of cells with centrosome aberrations decreased significantly at PD85 (X^2^, *p* < 0.05) (Figure 7C), concomitant with the reduction of polyploid cells most probably due to the impossibility of overcoming the p53 activation triggered by tetraploidy [29]. In contrast, attenuation of centrosome abnormalities in the diploid fraction of cells smoothed gradually over PDs (Figure 7C). Although it was not feasible to co-stain the cells for centrosome and senescent/apoptotic markers, there appeared to be a selection against diploid cells showing centrosome abnormalities. Notably, it has been described that centrosome amplification leads to an elevation of p53 levels and a corresponding decrease in proliferation of non-transformed cells [30]. Altogether, these data indicate a cooperative interaction of p16^INK4a^-deficiency and telomere-dependent DNA damage in the generation of centrosome aberrations in vHMECs.

To further confirm this connection, DNA-damage was induced in the p16^INK4a^-deficient vHMEC-hTERT by exposing the cells to ionizing radiation. vHMEC-hTERT at PD170 were irradiated under a cesium device and 48 hours later centrosome damage was evaluated. Genotoxic stress, in the form of DSBs, once again increased centrosome aberrations in vHMEC-hTERT (3.7% *vs* 9.5%, X^2^, *p* < 0.05) (Figure 7D), confirming the presence of anomalies in both number and size. In addition, CEP170/centrin immunofluorescence showed a significant increase in both overduplication (1.6% *vs* 5.3%, X^2^, *p* < 0.05) and accumulation of centrioles (1.1% *vs* 3.3%, X^2^, *p* < 0.05) in the irradiated cells. A somewhat higher proportion of cells with > 2CT was observed after radiation-induced genotoxic stress than after extensive telomere dysfunction (20% in irradiated vHMEC-hTERT *vs* 12.2% in post-stasis vHMEC at late PD, X^2^, *p* < 0.065), which could represent the premature disengagement and licensing effect of ionizing radiation [31]. Nevertheless, the induction of DNA damage in p16^INK4a^-deficient vHMEC-hTERT cells concluded again in a significant increase in centriole amplification events.

### Telomere-dependent instability activates the DNA damage checkpoint in proliferating vHMECs

A transient inhibition of DNA synthesis has been documented to be required for p16^INK4a^-deficient cells to generate more than two centrosomes in a Cdk2-dependent manner [21]. These authors demonstrated that in the absence of a functional p16^INK4a^ and upon hydroxyurea-induced DNA damage, p21^WAF1/CIP1^ cannot disengage from the cyclin D1/Cdk4/p21^WAF1/CIP1^ complex and thus completely inactivate cyclin E/A-Cdk2 [21]. Given that vHMECs proliferation is associated with the accumulation of uncapped telomeres, as well as the presence of double strand breaks [32], it is feasible that intrinsic DNA damage results in the activation of the DNA damage response (DDR), thus providing the opportunity for centrosome overduplication.

**Figure 8:**
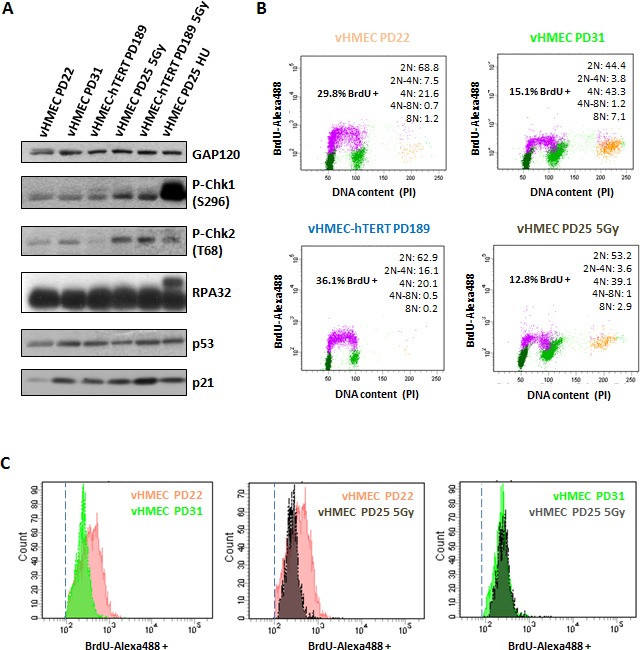
vHMECs accumulate DNA damage with PDs and slowdown DNA replication **A.** Western blot analysis of protein extracts from control and treated vHMEC and vHMEC-hTERT cells. vHMEC and vHMEC-hTERT cells were exposed to 5Gy of gamma rays and processed 10 hours post-irradiation. vHMECs treated with 4 mM HU during 6 hours were used as positive control for collapsed replication forks. GAP120 was used as loading control. **B.** Cell cycle analysis of vHMEC cells at different PDs, as well as irradiated vHMECs and vHMECs-hTERT, was performed by measuring BrdU-Alexa488 levels and propidium iodide staining. BrdU-Alexa488 positive cells are colored in purple, dark green represents cells with 2N DNA content, pale green represents 4N DNA content cells, and the yellow dots are cells with 8N DNA content. Legend (upper right) shows the distribution of cells with different DNA content. **C.** Histograms showing intensity levels of BrdU-Alexa488 positive vHMECs throughout PDs as well as irradiated vHMECs. Dashed line indicates the cut-off for BrdU positive cells.

To test this, western blot analyses were conducted in 440212 vHMECs to examine the expression of proteins associated with genotoxic damage throughout the cell culture. These experiments confirmed the results of previous studies, showing expression of wild-type p53 and p21 in cells lacking p16^INK4a^ expression [33-35]. No Chk1 or RPA-32 phosphorylation were observed in finite vHMEC, or in early or late PDs, indicating that there were not stalled or collapsed replication forks in these cells (Figure 8A). In contrast, phosphorylation of Chk2 and expression of p53 and p21^WAF1/CIP1^ were shown to increase with PDs in finite vHMEC (Figure 8A). Furthermore, irradiation of these cells induced only a slight increase in Chk2 phosphorylation that did not result in an increment in p53 and p21^WAF1/CIP1^ protein levels. Collectively, activation of Chk2-p53-p21 pathway throughout the cell culture indicates the accumulation of DNA damage in proliferating vHMECs. Next, we investigated whether vHMEC-hTERT also exhibit elevated levels of DDR proteins. Considering that telomere-dependent damage is abolished through telomerase expression, we expected a reduction of endogenous genotoxic damage and absence of DDR activation in hTERT stably expressing cells. Indeed, Chk2 phosphorylation on Thr68 was negligible in immortalized vHMECs. Moreover, although these cells have higher basal p21^WAF1/CIP1^ and p53 expression, upon irradiation they show Chk2 phosphorylation and a further increase in p53 and p21^WAF1/CIP1^ levels. In addition, no Chk1 or RPA-32 phosphorylation was observed unless HU was added (Figure 8A). Therefore, these data strongly support that proliferating vHMECs activate the Chk2-p53-p21 cascade with increasing PDs due to the accumulation of DSBs, probably arising from telomere uncapping.

We then aimed to assess whether finite vHMEC cells in late PDs also showed cell cycle arrest in association with the DDR activation. Cell cycle progression was monitored by FACS following a 30 minutes pulse with BrdU and propidium iodide staining. Proliferation of finite vHMECs resulted in a marked reduction of G1 cells (68.8% *vs* 44.4%, 2N fraction in the cytometer) and an increase of G2/M cells (21.6% *vs* 43.3%, 4N fraction in the cytometer) with PDs (Figure 8B). These results suggest that, similarly to irradiation, endogenous DNA damage observed in late vHMECs leads to the activation of the G2/M-checkpoint. However, the rise in the 8N fraction of cells at late PDs, and the already reported accumulation of cyclin D1 positive tetraploid vHMECs in the 4N DNA content window [9], did not allow to unambiguously determine the percentage of diploid cells corresponding to G2/M arrested cells. Nevertheless, the shift in BrdU intensity of late PD cells compared to early PD ones may indicate a slowdown in replication in late PD cells in response to telomere-dependent DNA damage. Consistent with this, a similar shift of BrdU incorporation was obtained in vHMECs after IR-induced genotoxic stress (Figure 8C). Taken together, all these data indicate that vHMECs cells accumulate DNA damage with PDs, in correlation with a delay in cell cycle progression in the S and G2/M phases.

## DISCUSSION

Almost all breast tumors (80-100%) display centrosome amplification [36]. In breast carcinogenesis, there is a significant correlation between centrosome aberrations and advancing disease [4]. Indeed, adenocarcinoma cells present abnormal numbers of centrosomes per cell; oversized centrosomes and/or centriole amplification, which are also observed to a lesser extent in pre-invasive *in situ* ductal carcinoma cells and/or pre-malignant breast lesions, thus revealing their premature onset in breast carcinogenesis [14, 36, 37]. Attempts to uncover the underlying origin of centrosome abnormalities in mammary carcinogenesis have determined a positive correlation between centrosome size and number defects with aneuploidy and CIN that is independent of p53 inactivation [14-16].

But how can centrosome defects arise early in breast carcinogenesis independently of p53 deficiencies? One possibility could be through targeting the pRB/p16^INK4a^ axis, an important pathway that modulates the proliferative lifespan of epithelial cells at the G1 boundaries and regulates the timely expression of a plethora of genes involved in centrosome homeostasis. Disruption of the pRB/p16^INK4a^-pathway occurs commonly in breast cancer. Loss of heterozygosity at the RB gene locus has been determined to be around 20-30% [38, 39] and overexpression or amplification of the cyclin D1 gene is observed in as many as 50% of cases [40]. Yet another mode of inactivation, with a prevalence ranging from 4% to 68.4%, is through *CDKN2A* promoter methylation [41-43]. This feature has been observed in various intraductal proliferative lesions as well as in high grade intraductal carcinomas [42]. Moreover, *CDKN2A* promoter methylation has been associated with a 6.58-fold increase of breast cancer risk [44] and occurs frequently in mammary cells obtained from women with greater chances of developing breast cancer [45, 46]. To unravel the importance of p16^INK4a^ in the generation of centrosome amplification early in breast cancer development, we made use of p16^INK4a^ proficient (pre-stasis HMEC) and deficient (vHMEC) human mammary epithelial cells. Our data provide evidence that p16^INK4a^-deficent vHMECs acquire increased numbers of centrosomes as well as centrosomes of greatly increased size that contain excessive numbers of centrioles concomitant to the accretion of telomere-dependent instability.

There is some controversy over the involvement of the pRB/p16^INK4a^ pathway in the origin of centrosome amplification. Leading studies have relied on the cell-cycle deregulation potential of human papilloma virus (HPV16) oncoproteins. The E7 oncoprotein has specifically been shown to induce prompt abnormal centrosome synthesis -seen as increased procentriole formation through electron microscope analysis -before the development of extensive nuclear abnormalities [47, 48] and polyploidization events [49]. HPV16 E7-expressing cells show a prolonged S-arrest, where an increased formation of immature daughter centrioles is produced [28] by a process similar to the one operating in hydroxyurea-treated cells [50]. Indeed, concurrent formation of more than one daughter centriole in single maternal templates, i.e. a rosette-like pattern, within a single S-phase, has been observed in cells expressing HPV16 E7 [51]. Nevertheless, any role of E7 oncoprotein in centrosome amplification must take into account the overwhelming targets with which this oncoprotein interacts, as it binds and degrades pRb, inactivates p21 and deregulates the expression of cyclin E, among others. In an attempt to better clarify this question, ectopic Cdk4 overexpression has been induced in immortalized keratinocytes (NOK-hTERT), given that it interferes with the inhibitory effect of p16^INK4a^ and results in increased pRB phosphorylation levels [52]. In that study, in striking contrast to what was observed in NOK-hTERT transduced with HPV16 E7, overexpression of Cdk4 in NOK-hTERT did not alter the centrosome duplication cycle [52], thus reflecting that abrogation of the pRb/p16^INK4a^ checkpoint does not necessarily lead to centrosome abnormalities. Furthermore, and in accordance with these observations, downregulation of p16^INK4a^ protein expression using specific shRNA in pre-stasis HMECs only resulted in a significant increase in centrosome amplification when cells were treated with hydroxyurea, an inhibitor of ribonucleotide reductase activity that induces replication stress DNA damage [21]. In the study concerned, centriole pair splitting was determined to be the main cause of centrosome aberrations in p16^INK4a^-deficient cells [21]. In addition, the same was found to occur in human mammary and newborn dermal foreskin fibroblasts transduced with p16^INK4a^ shRNA when exposed to hydroxyurea [21]. As a whole, all these studies support the notion that p16^INK4a^ prevents centrosome dysfunction when DNA damage is inflicted.

Given that telomere dysfunction is able to trigger a DNA damage response, it has been speculated that telomeric erosion may affect the status of the centrosome and even affect its amplification under the influence of DNA damage signaling [53]. In pre-stasis HMECs, inactivation of p16^INK4a^ allows cells to bypass senescence and display proliferative-dependent telomere dysfunction that ends up in uncapped chromosomes [20, 32]. Moreover, during proliferation, p16^INK4a^-deficient vHMECs accumulate uncapped DNA ends, as well as proper DSBs resulting from the entrance of end-to-end fusions into BFB-cycles [32]. Therefore, it could be considered that telomere-driven endogenous genotoxic stress could well be responsible for the centrosome amplification signature seen in telomere-compromised p16^INK4a^-deficent vHMECs. Indeed, our results illustrate that centrosome amplification events increased in parallel with intensification of DNA damage, probably arising from telomere uncapping, and restoration of telomere length by hTERT transduction concluded in the attenuation of the aberrant centrosome phenotype in diploid and polyploid cells. In agreement with these observations, disruption of the telomeric t-loop, through siRNA of the shelterin proteins TRF1 or POT1, in p16^INK4a^-deficient U2OS cell line, also resulted in an increase in supernumerary centrosomes [53].

Of even greater interest, we observed that alternative routes for centrosome amplification exist in telomere-compromised p16^INK4a^-deficient vHMEC. Whereas telomere-dependent polyploidization events ended up in abnormal centrosome numbers per cell -centrosome accumulation -telomere dysfunction also resulted in enlarged centrosomes that contained excessive numbers of centrioles -indicative that centrosome overduplication events were taking place. Centrosome duplication, like chromosome duplication, is initiated by Cdk2 [50], and similar to chromosomal DNA, centrosomes possess an intrinsic mechanism that ordinarily prevents their reduplication within a single cell cycle [54]. However, the coupling between centrosome duplication and cell cycle progression is much less robust with respect to conditions that impair cell cycle progression, and this condition is exacerbated when DNA damage is inflicted in cells with cell cycle checkpoint deficiencies. Indeed, mammalian cells lacking proper checkpoints controls are able to reduplicate their centrioles upon transient S-phase arrest, likely due to increased cyclin E-Cdk2 activity [reviewed by 55]. Similarly, when vHMECs are exposed to HU, which triggers inhibition of the DNA replication cycle, the absence of p16^INK4a^ allows the duplicated centrosomes to progress into a second round of centrosome duplication due to unregulated Cdk2 activity [21]. According to this model, the kinase activity of the cyclin E/A-Cdk2 is not completely inhibited by the CDKI p21^WAF1/CIP1^ because, in the absence of p16^INK4a^ protein, it remains associated with the cyclin D1-Cdk4 complex [21]. Our results in telomere-compromised vHMECs fit well with this model as accumulation of uncapped telomeres and DSBs with PDs results in the activation of the Chk2-p53-p21 cascade and a delay in cell cycle progression that could end up in the centrin amplification events observed. Nevertheless, the model depicted by McDermott does not resolve what occurs when p21^WAF1/CIP1^ levels rise due to DDR activation. Yet another possible explanation for cyclin E-Cdk2 upregulation comes from studies using a mutant CDK2 that lacks inhibitory phosphorylation sites [56]. Experiments showed that mutant MEFs with elevated Cdk2 activity had elevated numbers of centrosomes compared to wild-type MEFs, and this phenotype increased extensively after UV-irradiation [56]. Importantly, the observance that centrosome numbers were essentially unaffected by knocking down p21^WAF1/CIP1^, led the authors conclude that the subpopulation of cyclin E-Cdk2 that participates in centrosome duplication is located at the centrosome, so that it is physically separated from the nuclear protein p21^WAF1/CIP1^ [56]. As a whole, uncoupling of chromosome and centrosome duplication cycles occurs especially after checkpoint-induced cell cycle arrest, likely because cyclin A-Cdk2 activation would be further delayed relative to cyclin E-Cdk2 [56].

Although further studies are warranted to explore precisely the molecular pathways through which disruption of the pRB/p16^INK4a^ axis leads to centriole amplification in the presence of telomere-dependent genotoxic stress, our work has uncovered a mechanistic link for the coincident detection of telomere erosion and centrosome aberrations early in cancer development, before TP53 mutations are gained. In addition, it supports the notion that unrestricted growth due to pRB/p16^INK4a^ deficiencies, which occur in early breast lesions, will engender concomitant telomere- and centrosome-driven chromosome instability that, accompanied by other permissive lapses in cell cycle checkpoints, may underpin the onset of mammary carcinogenesis.

## MATERIALS AND METHODS

### Cells, culture and treatments

Post-stasis variant human mammary epithelial cells (vHMECs) 830 and 440212 were obtained from Cell Applications Inc. (San Diego, CA, USA). Pre-stasis HMEC were established from reduction mammoplasty tissue in accordance with previously reported methods [57]. The patient signed a written consent form allowing their tissue to be used for biological research; this consent was obtained by the medical staff at the hospital prior to surgery. All work with human derived material was reviewed and approved by the Human Subjects Protection Committee of the Universitat Autònoma de Barcelona.

HMECs and vHMECs were cultured with serum-free MEpiCM medium supplemented with MEpiCGS and penicillin/streptomycin (all from ScienCell Research Laboratories, Carlsbad, CA, USA). Growth conditions were 5% CO_2_ and 37°C. Culture population doublings (PDs) were calculated using the formula: PD = PD_initial_ + log_2_ (N_final_/N_initial_), where N_initial_ is the number of viable cells plated, and N_final_ is the number of viable cells harvested. vHMECs are finite, and the ones used in this study had a proliferative potential of 35-38 PDs.

vHMECs expressing hTERT (vHMEC-hTERT) were generated by lentiviral transduction of 830 and 440212 vHMECs at PD21, following standard procedures. The hTERT lentivirus was purchased from the Viral Vector Facility of CNIC (Spain).

To induce binucleated cells and centriole accumulation, vHMECs-hTERT were treated for 24 hours with 6μg/ml cytochalasin B (Cyt-B, Sigma-Aldrich, Tres Cantos, Spain); centriole overduplication was induced in vHMECs-hTERT after treatment with 2mM hydroxyurea (HU, Sigma-Aldrich) for 48h. Generation of double strand breaks (DSB) was achieved by irradiating vHMEC-hTERT with 5Gy of γ-rays using an IBL-437C R-^137^Cs source.

### Antibodies

Primary antibodies used were mouse β-tubulin (1:1000, Sigma-Aldrich), rabbit pericentrin (1:2000, Abcam, Cambridge, UK), mouse pericentrin (1:2000, Abcam), rabbit centrin (1:2000, gift from R. Ohi), mouse CEP170 (1:2000, Life Technologies, Alcobendas, Madrid), and mouse NEDD1 (1:1000, Abnova, Taipei, Taiwan) and mouse BrdU (1:100, Santa Cruz Biotechnology, Heidelberg, Germany) for flow cytometric analysis. Secondary antibodies were anti-mouse Cy3 (1:1000, Amersham, GE Healthcare Europe GmbH, Barcelona, Spain), anti-rabbit Alexa 488, and anti-rabbit Alexa 568 (both at 1:500, Life Technologies).

### Western blotting

Proteins were extracted with CHAPS lysis buffer and centrifuged at 4°C with 20000 rcf. Protein extracts were quantified with NanoDrop 2000 (Thermo Fisher Scientific S.L., Barcelona, Spain) and denatured for 10 minutes at 70°C. The proteins were then separated using a 10% SDS-PAGE gel (Life Technologies), and transferred to a nitrocellulose membrane. Antibodies used were, mouse p16^INK4a^ (1:1000, Neomarkers, Fremont, CA, USA), mouse p53 (1:1000, Santa Cruz Biotechnologies, Heidelberg, GE) and mouse GAPDH (1:1000, Abcam) diluted in 1xPBS-3%BSA-0.1%Tween20. Horseradish peroxidase (HRP)-conjugate anti-mouse was used as secondary antibody (1:2000, Millipore, Madrid, Spain). Chemiluminescent detection of antibodies was performed using HRP solution and luminol (Immobilion Western kit, Millipore).

For P-Chk1 (S296), P-Chk2 (T68), p53, p21 and RPA32 protein detection (Figure 8), previously treated samples were collected in 2% SDS containing 67 mM Tris-HCl (pH 6.8) buffer. Protein extracts were denatured for 15 minutes at 95°C and quantified by common methods. The proteins were then run in Laemmli SDS-polyacrylamide gels and transferred to nitrocellulose membranes before their detection with the indicated antibodies: rabbit P-Chk1 S296 (1:1000, Cell signaling, #2349), rabbit P-Chk2 T68 (1:1000, NB100-92502), mouse p53 (1:1000, Ab-5, MS-186), mouse p21 (1:1000, Ab-1, OP64), rat RPA32 (1:1000, Cell signaling, #2208) and mouse GAP120 (1:400, santa cruz, sc-63), diluted in 1xPBS-3%BSA-0.05%Tween20. Horseradish peroxidase (HRP)-conjugate antibodies were used as secondary antibodies. Proteins were finally visualized using ECL detection system (Biological Industries).

### Immunofluorescence

Cells plated onto chamber slides were fixed with ice-cold methanol for 3 minutes when 70%-80% confluence was reached. The cells were then permeabilized with 1xPBS-1%Triton-X-100 solution for 15 minutes and blocked with 1xPBS-0.1%Tween20-2% Fetal Calf Serum or 1xPBS-5%BSA for 1 hour at 37°C. When centrin antibodies were specifically used, cells were incubated in KCM buffer (120mM KCl, 20mM NaCl, 10mM Tris-HCl -pH 8-, 0.5mM EDTA and 0.1% Triton X-100) prior to the permeabilization step. Incubation with primary antibodies diluted in blocking solution was performed for 1 hour at 37°C, and secondary antibodies were incubated for 30 minutes in blocking solution at room temperature. When centrin antibodies were used, washes between different steps were in 1xPBS or PHEM buffer (60mM PIPES, 25mM HEPES, 20mM EGTA, 2mM MgCl_2_). Finally, after secondary antibody incubation, samples were washed with 1xPBS-0.5%Tween20, dehydrated and counterstained with 4′,6-diamidino-2-phenylindole (DAPI).

### ImmunoFISH

ImmunoFISH protocol consists of performing immunofluorescence first, as described in the previous section, except for the final steps. After washing for excess secondary antibody with 1xPBS-0.5%Tween20 the samples were fixed again using a microtubule stabilization and extraction fixative buffer (MTSB-XF; 100mM PIPES, 5mM MgCl_2_, 2.5mM EGTA, 1mM DTT, 1μM taxol, 0.01%, aprotinin, 50% deuterium oxide, 3.7% formaldehyde, 0.1% Triton X-100) for 5 minutes at 37°C and fixed with 4% formaldehyde and then dehydrated. DNA denaturation was then achieved in 2xSSC-70% formamide for 10 minutes at 73°C and followed by cold ethanol dehydration. For Pericentrin/oligoFISH analysis, the centromere of chromosome 6 (Gold DY539) and 12 (Red DY590) oligonucleotide specific probes (OligoFISH Probes, Cellay Inc., Cambridge, MA, USA) were hybridized with the denatured samples overnight at 37°C. Afterwards, the samples were briefly washed in 0.2xSSC-0.1%SDS at 50°C. For the NEDD1/FISH analysis, CEP 4 and LSI 22 (bcr) DNA probes (Abbot, Abbot Park, IL) were denatured for 5 minutes at 74°C and afterwards hybridized with the denatured samples at 37°C overnight. Afterwards, the samples were washed with 0.4xSSC at 73°C for 1 minute and 2xSSC 0.5%Tween20 at room temperature for 1 minute. All slides were counterstained with DAPI after dehydration in graded series of alcohol.

### Microscope analysis and size measurement

Fluorescence was evaluated under an optical epifluorescence microscope with specific filters for each of the used fluorochromes, and images were obtained using Isis Fluorescence Imaging software (MetaSystems GmbH, Altlussheim, DE), or Genus software (Cytovision, Leica Microsystems S.L.U., Barcelona, Spain).

Centrosome size measurement was performed in vHMEC-hTERT using antibodies against pericentrin, which label the pericentriolar material, and centrin, to visualize individual centrioles. The raw images of each single fluorochrome capture were processed with the Fiji package (http://fiji.sc/Fiji) [58], using a ROI management tool.

### Flow cytometric analysis

For tracking S phase cells, a pulse of 10μM BrdU was carried out for 30 minutes. Afterwards, cells were rinsed, trypsinized, centrifuged and fixed with 70% ethanol. The samples were kept at −20°C until processing. Detection of BrdU incorporation was achieved following standard procedures after DNA denaturation with HCl 2N-0.5%Triton-X-100. Before antibody detection, every sample was divided into two different tubes. One of the tubes was incubated with both anti-BrdU primary antibody and anti-mouse Alexa 488, the control tub was only incubated with the secondary antibody. Before acquiring the samples, they were counterstained with 0.5% Propidium Iodide (PI; 1mg/ml) in 1xPBS-0.1%TritonX100 containing 0.2 mg/ml RNAase A DNAase-free (Sigma-Aldrich). Cell-cycle analysis was performed in a FACSCalibur. All results were analyzed with the BDFacsDiva software (BD Biosciences).

### Statistical analysis

Data analysis was carried out with the statistical program SPSS 15.0 for Windows (SPSS Inc, University of Chicago). Statistical differences between the samples analyzed were considered to be significant when a *p*-value < 0.05 was obtained.

## SUPPLEMENTARY MATERIAL FIGURES



## Abbreviations

BFB: Breakage-Fusion-Bridge
BN: Binucleated cells
Cdk4: Cyclin-dependent kinase 4
CDKN2A: Cyclin-dependent kinase inhibitor 2A gene
HPV16 E7: Human papillomavirus type 16 E7 oncoprotein
hTERT: Human telomerase reverse transcriptase
MEF: Mouse embryonic fibroblast
NOK-hTERT: Immortalized Keratinocytes
p16^INK4A^: Cyclin-dependent kinase inhibitor 2A protein
p21^CIP1^: Cyclin-dependent kinase inhibitor 1
p27^KIP1^: Cyclin-dependent kinase inhibitor 1B
p53: Cellular tumor antigen p53 protein
pRB: retinoblastoma-associated protein
TP53: Cellular tumor antigen p53 gene
vHMEC-hTERT: Immortalized variant human epithelial cells
vHMEC: Variant human mammary epithelial cells
