# Supplementary material for: Centrosome aberrations in human mammary epithelial cells driven by cooperative interactions between p16INK4a deficiency and telomere-dependent genotoxic stress
Source: Oncotarget. 2015 Jul 22;6(29):28238–56. doi: 10.18632/oncotarget.4958 (PMC4695057; doi:10.18632/oncotarget.4958)
Supplement: Supplementary file 1 [file oncotarget-06-28238-s001.pdf]

Centrosome aberrations in human mammary epithelial cells driven by cooperative interactions between p16^{INK4a} deficiency and telomere-dependent genotoxic stress

Supplementary Material

PNA-FISH

Chromosome spreads of vHMEC-hTERT were obtained after colcemid (Life Technologies) treatment, swelling of cells in 0.075M potassium chloride at 37^{0}C, and fixation with methanol/acetic acid (3:1).Slides containing metaphase spreads were briefly treated with pepsin/HCl at 37^{0}C, rinsed in 1xPBS and post-fixed with formaldehyde-$MgCl_2$. The hybridization mix containing a pancentromeric PNA-FITC probe and a pantelomeric PNA-Cy3 probe (PE Biosystems, Framingham, MA, USA) was dropped onto slides and denatured at 80^{0}C for 90 seconds. Hybridization was performed at room temperature for 2 hours, followed by post- hybridization washing steps with 70% formamide and Tris-NaCl-Tween20 buffer (TNT). Finally, slides were dehydrated and mounted in antifade solution containing 0.125 µg/ml DAPI before proceeding to the microscopic analysis.

Texas Red-X phalloidin staining

Cells grown in chamberslides were fixed with 4% paraformaldehyde for 10 minutes. The cells were then permeabilized with 1xPBS-1%Triton-X-100 solution for 10 minutes at room temperature. Afterwards, Texas Red-X phalloidin staining (Life Technologies) was diluted with blocking solution at a final concentration of 1.5 U/ml and applied for 5 minutes. Finally, samples were washed with 1xPBS, dehydrated and counterstained with DAPI.

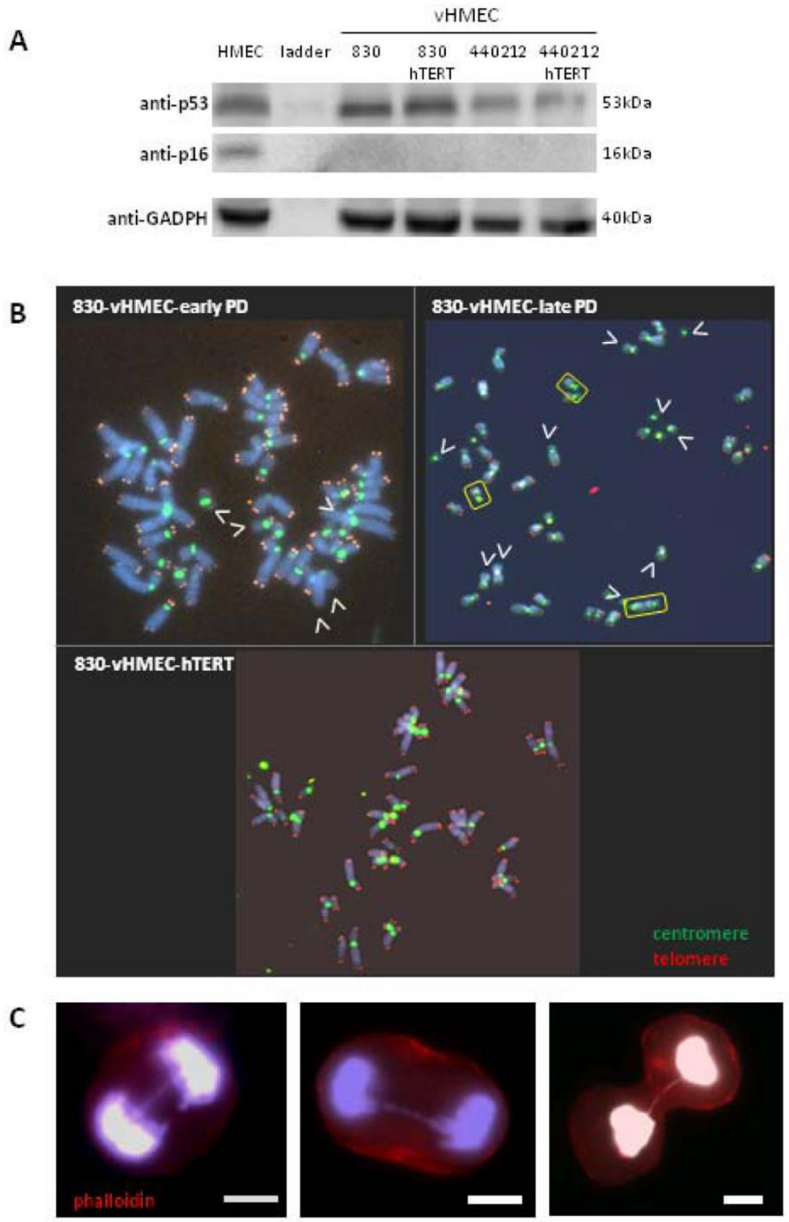

Figure S1: Characterization of pre-stasis HMECs and post-stasis vHMEC and vHMEC-hTERT

(A) Western blot analysis of p53 and p16 in pre- and post-stasis HMEC. Proteins were extracted at PD:3 (pre-stasis HMEC); PD:26 (830 vHMEC) and PD:141 (830 vHMEC-hTERT); and PD:23 (440212 vHMEC) and PD:137 (440212 vHMEC-hTERT). Whereas only pre-stasis HMEC show p16^{INK4a} expression, all types of cells analyzed showed p53 expression. GADPH antibody was used as loading control.

(B) Metaphase spreads of 830 vHMECs, at different PDs, and 830 vHMECs-hTERT were obtained after colcemid addition. Subsequent PNA-FISH allowed the identification of telomere dysfunction hallmarks, i.e. chromosomes without telomeric signal —pointed arrows— and end- to-end chromosome fusions —yellow squares. On the first row, 830 vHMEC metaphases at early PD (right side) and late PD (left side). At the bottom, a representative

metaphase of vHMEC- hTERT. Chromosomes are stained with DAPI: the centromeric probe is green and the telomeric probe is red.

(C) Telomere dysfunction was also evaluated through the visualization of chromatin bridges during cell division. Cells were grown on chamberslides and stained with Texas-red X phalloidin (actin cortex) and DAPI (DNA). Representative images of anaphase bridges in 440212 vHMEC at late PD, during the progression of ana-telofase and cytokinesis. White bars represent 5 µm.

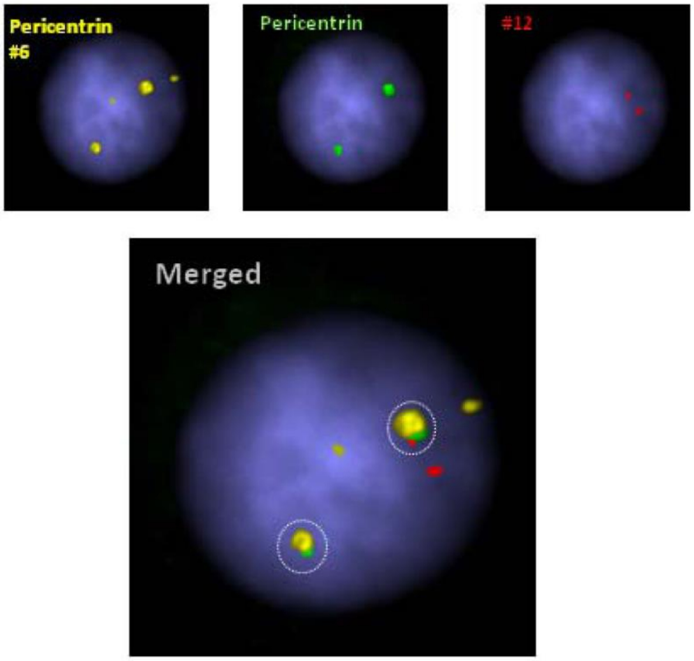

Figure S2. ImmunoFISH protocol enabled identification of the ploidy status of cells with and without centrosome aberrations.

Representative example of a mononucleated diploid cell showing two centrosomes. Each small micrograph is a composite of a single fluorochrome capture and DAPI staining. First image shows two big spots that correspond to the pericentrin signal —detected with anti-mouse- Cy3— and two tiny signals for the centromere of chromosome 6 —labeled with gold DY539. Second image shows the two spots of pericentrin detected with anti-mouse-Alexa488. Third image shows the two tiny signals of the centromere of chromosome 12 —labeled with red DY590. In the merged image (bottom) the double-fluorochrome labeled centrosomes are encircled by a dashed white line.

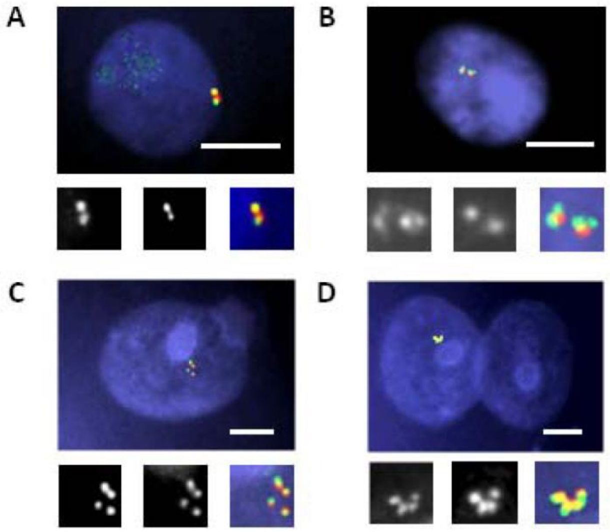

Figure S3. Normal pattern of NEDD1/centrin labeling in mononucleated and binucleated vHMECs.

Representative images of the most common signal patterns after NEDD1/centrin immunofluorescence.

(A) Mononucleated cell showing 2 centrin and 2 NEDD1 signals that fully colocalize.

(B) Mononucleated vHMEC showing 4 centrin signals and only 2 NEDD1 signals.

(C) Mononucleated cell showing 4 colocalizing centrin/NEDD1 signals.

(D) Binucleated –tetraploid- vHMEC showing 4 centrin and 4 NEDD1 colocalizing signals. At the bottom of each image, insets show individual centrin and NEDD1 signals and the merged image. Bar represents 5 μm.

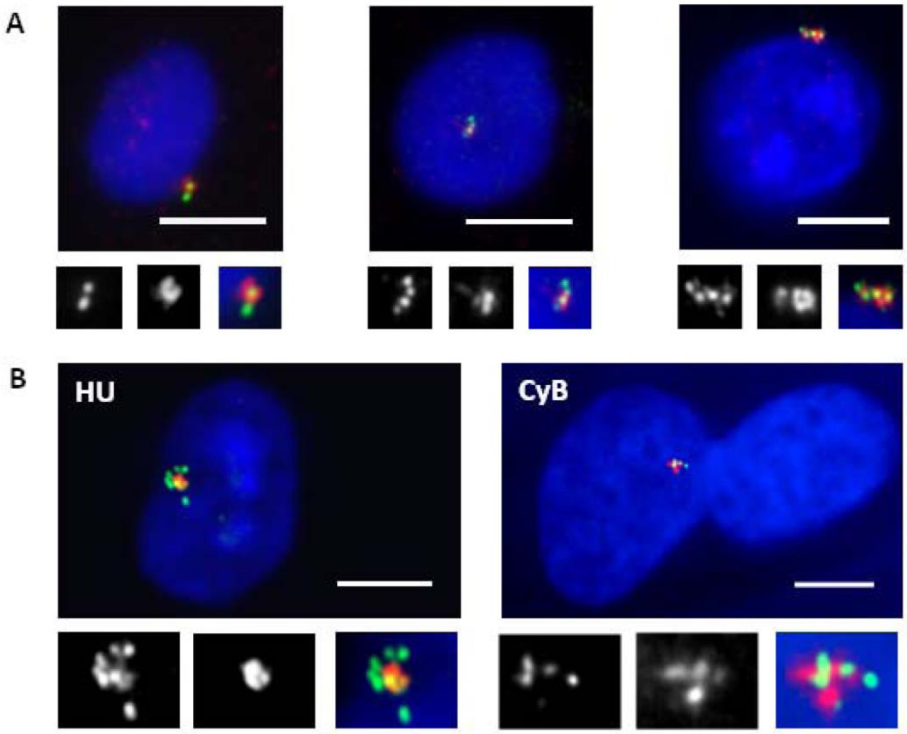

Figure S4. Pattern of centrin/CEP170 staining

To distinguish between mature and immature centrioles, centrin antibodies were combined with CEP170, a mature centriole marker. A normal centrosome was considered when no more than 4 centrioles were displayed, 1 or 2 of them being mature centrioles.

(A) Representative images of mononucleated cells with normal centrosomes: 2 centrin/1 CEP170 signal (left), 4 centrin/1 CEP170 signal (center) and 4 centrin/2 CEP170 signal (right). At the bottom of each image, insets show individual centrin and CEP170 signals and the merged image. Bar, 5 µm.

(B) In order to ascertain the amplification and accumulation phenotype with the defined antibodies, vHMEC-hTERT was treated with different drugs. Treatment with hydroxyurea (HU) resulted in overduplication of centrioles, and was visualized by the presence of more than 4 centrioles, 1 or 2 being mature centrioles. Representative image of overduplication on the left side, where around 7 centrin signals and only one CEP170 are observed. Cells were also treated with cytochalasin B (CytB) in order to abort cytokinesis and engender accumulation of the centrosomes in the cytoplasm. In this case, accumulation of centrosomes was considered when the cell showed more than 4 centrioles, 50% of them being mature. Representative example of a binucleated cell with accumulation of centrosomes. At the bottom of each image, insets show individual centrin and CEP170 signals and the merged image. Bar represents 5 µm.
